# Complete Annotated Genome Sequence of Limosilactobacillus fermentum AGR1487

**DOI:** 10.1128/MRA.01056-20

**Published:** 2021-01-07

**Authors:** Marc A. Bailie, Eric Altermann, Wayne Young, Nicole C. Roy, Warren C. McNabb

**Affiliations:** a School of Food and Advanced Technology, Massey University, Palmerston North, New Zealand; b Food Nutrition & Health Team, AgResearch, Grasslands Research Centre, Palmerston North, New Zealand; c Riddet Institute, Massey University, Palmerston North, New Zealand; d The High-Value Nutrition National Science Challenge, Auckland, New Zealand; e Department of Human Nutrition, University of Otago, Dunedin, New Zealand; f Liggins Institute, University of Auckland, Auckland, New Zealand; Loyola University Chicago

## Abstract

Limosilactobacillus fermentum is a probiotic species; however, *L. fermentum* AGR1487 increases colon inflammation in germfree mice and decreases barrier integrity in Caco-2 cells. The AGR1487 genome was sequenced to explore these phenotypes. The genome is a single, circular, 1,939,032-bp chromosome with a G+C content of 52.17% and no plasmids.

## ANNOUNCEMENT

Limosilactobacillus fermentum strains are regularly used for fermented food production and preservation as acid-producing starter cultures (1, 2). In humans, strains of *L. fermentum* have been shown to improve the ratio of beneficial microorganisms of the large intestine and have been used as probiotic treatments for intestinal and vaginal diseases (1–3). However, *L. fermentum* AGR1487 has been found to increase colon inflammation in germfree mice and decreased the barrier integrity of Caco-2 monolayers (4, 5). *L. fermentum* AGR1487 was isolated from an oral swab of a healthy human and identified using 16S rRNA gene sequencing (6). AGR1487 was sequenced to explore this unique barrier disruptive phenotype and its genetic characteristics.

*L. fermentum* AGR1487 cells were grown in de Man-Rogosa-Sharpe (MRS) broth (Merck Ltd., Auckland, New Zealand) to stationary phase overnight at 37°C. Genome extraction, purification, and Illumina and PacBio shotgun sequencing were carried out as previously described (7). The Illumina library was created using the TruSeq library kit with genomic DNA sheared into 500-bp fragments and sequenced on a HiSeq 2000 genome analyzer. Illumina sequencing generated 2,523,872 2 × 100-bp paired-end (200-bp combined) Illumina reads. The sheared genomic DNA was used for the creation of a 10-kb PacBio SMRTbell library. Ten-kilobyte size selection conditions were used when purifying the hairpin dimers by magnetic bead, and the adapters were removed using PacBio’s MagBead kit. Sequencing was carried out on the PacBio Sequel platform, generating 344,060 subreads with an average length of 8,498 bp and an *N*_50_ value of 9,837 bp.

Default parameters were applied for all software packages unless otherwise specified. Illumina short-read quality control was done using FastQC v0.11.9 (8) before and after trimming with Trimmomatic v0.39 (9). Assembly graphs were assessed for errors using Bandage v0.8.1 (10). A single circular genome assembly was produced by Unicycler v0.4.7 (11) using the trimmed Illumina short reads along with uncorrected PacBio long reads. The final genome assembly was polished for three rounds using Pilon v1.22 (12).

**FIG 1:**
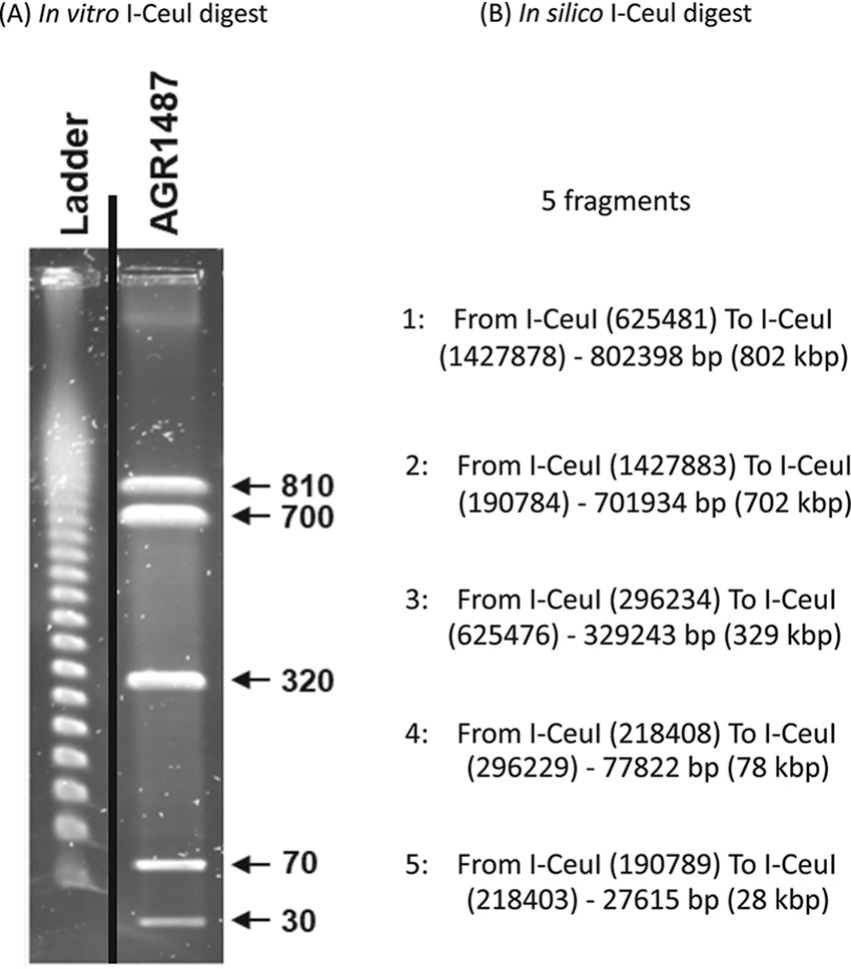
(A) Pulsed-field gel electrophoresis of AGR1487 genomic DNA digested with restriction enzyme I-CeuI. The marker ladder contained lambda DNA, where the fragments were multimers of 48.5 kb. The values given are the sizes (kb) of the DNA fragments from the bacterial strains. The graph depicts the relevant parts of the original published gel image for conciseness, and the vertical black line indicates the boundaries between image slices. (Adapted from *Microbiologyopen* [4].) (B) *In silico* digest results of the AGR1487 genome assembly using I-CeuI restriction sites processed by UGENE (11) and presented as the range from one restriction site to the next (fragment size in bp and Kbp).

CheckM v.1.0.18 (13) reported genome completeness rates of 99.18% and 0.55% contamination. The basic statistics were calculated using QUAST v4.6.3 (14), which found that the resulting assembly was a single 1,939,032-bp contig with a G+C content of 52.17% and no ambiguous bases or gaps filled with arbitrary place holders (Ns). The expected average read depth was calculated to be 1,510.51×. The genome assembly was uploaded to UGENE v34.0 (15), the ends of the sequence were digitally overlapped, and an *in silico* digest at the I-CeuI restriction sites was calculated. The resulting fragment pattern from the *in silico* digest matched a previously published restriction digest of AGR1487 that used a commercial I-CeuI restriction enzyme (Fig. 1) (4).

PGAP v4.10 (16) and GAMOLA2 v16.0 (17) were used to annotate the AGR1487 genome assembly, which was found to harbor 2,065 open reading frames (ORFs), 1,743 conserved domains, and 1,666 clusters of orthologous groups (COGs). The genetic origin of the barrier disruptive phenotype for this strain is likely found in this chromosome, as no plasmids were found during genomic DNA purification and sequence assembly.

### Data availability.

The PacBio long reads and Illumina MiSeq sequence reads described here have been deposited at NCBI/GenBank under BioProject accession number PRJNA596816. The whole-genome sequence is available from NCBI/GenBank under BioSample accession number SAMN13639333 or directly using the assembly accession number CP047585.
